# 2-Benzoyl-1,1-diethyl-3-phenyl­guanidine

**DOI:** 10.1107/S1600536809001469

**Published:** 2009-01-17

**Authors:** Ghulam Murtaza, Hanif-Ur-Rehman, M. Khawar Rauf, Masahiro Ebihara, Amin Badshah

**Affiliations:** a Department of Chemistry, Quaid-i-Azam University Islamabad, 45320-Pakistan; b Institute of Chemical Sciences, University of Peshawar, Peshwar-Pakistan; c Department of Chemistry, Faculty of Engineering, Gifu University Yanagido, Gifu 501-1193, Japan

## Abstract

In the title tetrasubstituted guanidine, C_18_H_21_N_3_O, the guanidine and carbonyl groups are not coplanar, as reflected by the torsion angles involving the N=C atoms [17.6 (3), −141.68 (17) and 42.2 (3)°]. This is probably due to the absence of an intra­molecular N—H⋯O hydrogen bond, forming a six-membered ring, and is commonly observed in this class of compounds. In the crystal structure, centrosymmetric dimers are formed *via* pairs of inter­molecular N—H⋯O hydrogen bonds. The dihedral angles between the guanidine plane and the phenyl ring and benzoyl plane are38.06 (9) and 41.54 (7)°, respectively.

## Related literature

For thio­urea derivatives with biological activity, see: Berlinck (2002 ▶); Heys *et al.* (2000 ▶); Laeckmann *et al.* (2002 ▶); Kelley *et al.* (2001 ▶); Moroni *et al.* (2001 ▶); Ishikawa *et al.* (2002 ▶). For related structures, see: Murtaza *et al.* (2007 ▶, 2008 ▶); Cunha *et al.* (2005 ▶).
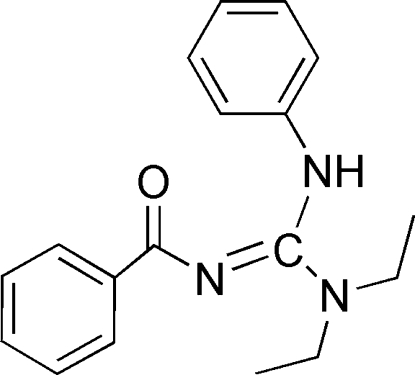


## Experimental

### 

#### Crystal data

C_18_H_21_N_3_O
*M* *_r_* = 295.38
Monoclinic, 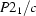

*a* = 10.472 (6) Å
*b* = 15.010 (8) Å
*c* = 10.154 (6) Å
β = 102.992 (6)°
*V* = 1555.2 (15) Å^3^
*Z* = 4
Mo *K*α radiation
μ = 0.08 mm^−1^
*T* = 113 (2) K
0.50 × 0.40 × 0.30 mm

#### Data collection

Rigaku/MSC Mercury CCD diffractometer
Absorption correction: none
12318 measured reflections
3556 independent reflections
3239 reflections with *I* > 2σ(*I*)
*R*_int_ = 0.042

#### Refinement

*R*[*F* ^2^ > 2σ(*F* ^2^)] = 0.068
*wR*(*F* ^2^) = 0.112
*S* = 1.27
3556 reflections
205 parameters
H atoms treated by a mixture of independent and constrained refinement
Δρ_max_ = 0.26 e Å^−3^
Δρ_min_ = −0.19 e Å^−3^

### 

Data collection: *CrystalClear* (Molecular Structure Corporation & Rigaku, 2001 ▶); cell refinement: *CrystalClear*; data reduction: *TEXSAN* (Molecular Structure Corporation & Rigaku, 2004 ▶); program(s) used to solve structure: *SIR97* (Altomare *et al.*, 1999 ▶); program(s) used to refine structure: *SHELXL97* (Sheldrick, 2008 ▶); molecular graphics: *ORTEPII* (Johnson, 1976 ▶); software used to prepare material for publication: *SHELXL97* and *TEXSAN*.

## Supplementary Material

Crystal structure: contains datablocks I, global. DOI: 10.1107/S1600536809001469/su2090sup1.cif

Structure factors: contains datablocks I. DOI: 10.1107/S1600536809001469/su2090Isup2.hkl

Additional supplementary materials:  crystallographic information; 3D view; checkCIF report

## Figures and Tables

**Table 1:** Hydrogen-bond geometry (Å, °)

*D*—H⋯*A*	*D*—H	H⋯*A*	*D*⋯*A*	*D*—H⋯*A*
N3—H3⋯O1^i^	0.90 (2)	1.97 (2)	2.852 (2)	168 (2)

Symmetry code: (i) 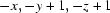.

**Fig. 1.**
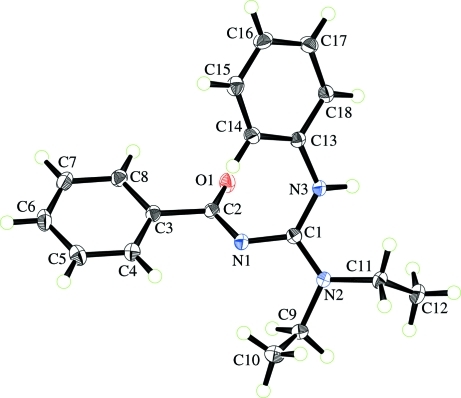
Molecular structure of compound (I), showing the atom numbering scheme. Displacement ellipsoids are drawn at the 50% probability level.

**Fig. 2.**
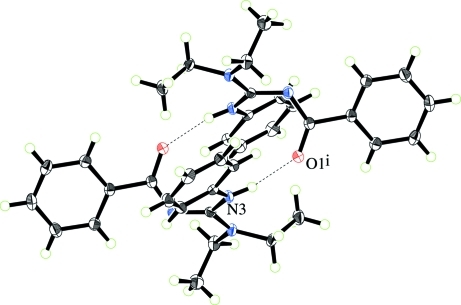
A view of the centrosymmetric hydrogen-bonded dimer structure of compound (I) [Hydrogen bonds shown as dashed lines; symmetry code: (i) -x, 1 - y, 1 - z].

## supplementary crystallographic information

### Comment

Guanidines are important compounds that have many biological, chemical and medicinal applications (Berlinck *et al.*, 2002; Heys *et al.*, 2000). They have received increasing interest as medicinal agents with antitumour, anti-hypertensive, anti-glaucoma and cardiotonic activities (Laeckmann *et al.*, 2002; Kelley *et al.*, 2001; Moroni *et al.*, 2001). Due to their strongly basic character, they can be considered as super-bases that readily undergo protonation to generate resonance-stabilized guanidinium cations (Ishikawa *et al.*, 2002).

The molecular structure of the title compound, (I), is illustrated in Fig. 1. It is a typical *N',N,N,N''*-Tetrasubstituted guanidine with normal geometrical parameters (Murtaza *et al.*, 2007, 2008; Cunha *et al.*, 2005). The carbonyl bond (C2═O1) shows the expected full double bond character, while the shorter values for bonds C2—N1, N1-C1, C1—N2, and C1—N3 indicate partial double bond character. The dihedral angles between the guanidine mean plane (C1/N1/N2/N3), and the phenyl ring (C13–C18), the benzoyl ring (C3-C8,C2,O1) and the N2/C9/C11 plane, are 38.06 (9)°, 41.54 (7)°, and 11.97 (13)°, respectively. The guanidine moiety and the carbonyl group are not co-planar, as reflected by the torsion angles C1—N1—C2—O1 = 17.6 (3)°, N2—C1—N1—C2 = -141.68 (17)°, and N3—C1—N1—C2 = 42.2 (3)°. This is probably due to the absence of an intramolecular N—H···O hydrogen bond, forming a six-membered ring, and commonly observed in this class of compounds (Cunha *et al.*, 2005).

The crystal packing shows intermolecular N—H···O hydrogen bonds which result in the formation of centrosymmetric dimers (Fig. 2).

### Experimental

*N*-Benzoyl-*N'*-phenylthiourea (0.512 g, 2 mmol), triethyl amine (0.56 ml, 4 mmol) and diethyl amine (0.11 mL, 2 mmol) dissolved in 20 ml dimethylformamide, were mixed with vigourous stirring at 5°C. Mercuric chloride (0.544 g, 2 mmol) was then added and the mixture vigorously stirred for 12 h. The progress of the reaction was monitored by TLC. When all the thiourea had been consumed, 20 mL of chloroform were added and the suspension was filtered through a cintered glass funnel to remove any residue (HgS) formed as a byproduct during the reaction. The solvent was evaporated under reduced pressure and the residue was dissolved in 20 mL of CH_2_Cl_2_. Other byproducts were extracted out with water (4× 30 mL). The organic phase was dried over anhydrous MgSO_4_ and then filtered. After filtration the solvent was evaporated and compound (I) was recrystallized in ethanol. Full spectroscopic and physical characterization will be reported elsewhere.

### Refinement

The N-H hydrogen atom was located in a difference Fourier map and freely refined: N-H = 0.90 (2) Å. The C-bound H-atoms were included in calculated positions and treated as riding atoms: C—H = 0.95 - 0.98 Å with *U*_iso_(H) = 1.2 or 1.5*U*_eq_(C).

### Crystal data

**Table:**

C_18_H_21_N_3_O	*F*(000) = 632
*M**_r_* = 295.38	*D*_x_ = 1.262 Mg m^−^^3^
Monoclinic, *P*2_1_/*c*	Mo *K*α radiation, λ = 0.71070 Å
Hall symbol: -P 2ybc	Cell parameters from 4060 reflections
*a* = 10.472 (6) Å	θ = 6.3–55.0°
*b* = 15.010 (8) Å	µ = 0.08 mm^−^^1^
*c* = 10.154 (6) Å	*T* = 113 K
β = 102.992 (6)°	Block, colourless
*V* = 1555.2 (15) Å^3^	0.50 × 0.40 × 0.30 mm
*Z* = 4	

### Data collection

**Table:**

Rigaku/MSC Mercury CCD diffractometer	3239 reflections with *I* > 2σ(*I*)
graphite	*R*_int_ = 0.042
Detector resolution: 14.62 pixels mm^-1^	θ_max_ = 27.5°, θ_min_ = 3.4°
ω scans	*h* = −13→8
12318 measured reflections	*k* = −19→19
3556 independent reflections	*l* = −13→13

### Refinement

**Table:**

Refinement on *F*^2^	Primary atom site location: structure-invariant direct methods
Least-squares matrix: full	Secondary atom site location: difference Fourier map
*R*[*F*^2^ > 2σ(*F*^2^)] = 0.068	Hydrogen site location: inferred from neighbouring sites
*wR*(*F*^2^) = 0.112	H atoms treated by a mixture of independent and constrained refinement
*S* = 1.27	*w* = 1/[σ^2^(*F*_o_^2^) + (0.0155*P*)^2^ + 1.0509*P*] where *P* = (*F*_o_^2^ + 2*F*_c_^2^)/3
3556 reflections	(Δ/σ)_max_ < 0.001
205 parameters	Δρ_max_ = 0.26 e Å^−^^3^
0 restraints	Δρ_min_ = −0.19 e Å^−^^3^

### Special details

**Table:**

Geometry. All e.s.d.'s (except the e.s.d. in the dihedral angle between two l.s. planes) are estimated using the full covariance matrix. The cell e.s.d.'s are taken into account individually in the estimation of e.s.d.'s in distances, angles and torsion angles; correlations between e.s.d.'s in cell parameters are only used when they are defined by crystal symmetry. An approximate (isotropic) treatment of cell e.s.d.'s is used for estimating e.s.d.'s involving l.s. planes.
Refinement. Refinement of *F*^2^ against ALL reflections. The weighted *R*-factor *wR* and goodness of fit *S* are based on *F*^2^, conventional *R*-factors *R* are based on *F*, with *F* set to zero for negative *F*^2^. The threshold expression of *F*^2^ > σ(*F*^2^) is used only for calculating *R*-factors(gt) *etc*. and is not relevant to the choice of reflections for refinement. *R*-factors based on *F*^2^ are statistically about twice as large as those based on *F*, and *R*- factors based on ALL data will be even larger.

### Fractional atomic coordinates and isotropic or equivalent isotropic displacement parameters (Å^2^)

**Table:**

	*x*	*y*	*z*	*U*_iso_*/*U*_eq_	
C1	0.21836 (16)	0.49684 (12)	0.59825 (17)	0.0158 (3)	
N1	0.28409 (14)	0.54983 (10)	0.53093 (14)	0.0174 (3)	
N2	0.24622 (14)	0.50194 (10)	0.73344 (14)	0.0172 (3)	
N3	0.12913 (15)	0.43370 (10)	0.53944 (14)	0.0171 (3)	
H3	0.063 (2)	0.4210 (15)	0.580 (2)	0.035 (6)*	
C2	0.22449 (17)	0.58833 (11)	0.41264 (16)	0.0162 (3)	
O1	0.10437 (12)	0.59798 (9)	0.36752 (12)	0.0208 (3)	
C3	0.31721 (17)	0.62748 (11)	0.33424 (17)	0.0168 (4)	
C4	0.45097 (18)	0.63592 (12)	0.39163 (18)	0.0201 (4)	
H4	0.4854	0.6146	0.4806	0.024*	
C5	0.53391 (19)	0.67529 (13)	0.31946 (19)	0.0248 (4)	
H5	0.6247	0.6810	0.3593	0.030*	
C6	0.4846 (2)	0.70640 (13)	0.18924 (19)	0.0249 (4)	
H6	0.5414	0.7337	0.1402	0.030*	
C7	0.35200 (19)	0.69752 (12)	0.13084 (18)	0.0228 (4)	
H7	0.3182	0.7184	0.0414	0.027*	
C8	0.26838 (18)	0.65820 (12)	0.20278 (17)	0.0195 (4)	
H8	0.1777	0.6522	0.1623	0.023*	
C9	0.32896 (18)	0.57493 (13)	0.80129 (18)	0.0222 (4)	
H9A	0.3065	0.5871	0.8892	0.027*	
H9B	0.3097	0.6294	0.7455	0.027*	
C10	0.47450 (19)	0.55474 (14)	0.82538 (19)	0.0286 (4)	
H10A	0.4928	0.4974	0.8718	0.043*	
H10B	0.5243	0.6018	0.8814	0.043*	
H10C	0.5003	0.5521	0.7385	0.043*	
C11	0.20739 (18)	0.43504 (12)	0.82325 (17)	0.0202 (4)	
H11A	0.2858	0.4169	0.8922	0.024*	
H11B	0.1734	0.3816	0.7694	0.024*	
C12	0.10328 (19)	0.46898 (14)	0.89432 (19)	0.0262 (4)	
H12A	0.1380	0.5199	0.9518	0.039*	
H12B	0.0793	0.4213	0.9502	0.039*	
H12C	0.0256	0.4876	0.8267	0.039*	
C13	0.12031 (17)	0.39230 (11)	0.41262 (16)	0.0156 (3)	
C14	0.22270 (18)	0.39048 (12)	0.34509 (17)	0.0195 (4)	
H14	0.3026	0.4205	0.3821	0.023*	
C15	0.20751 (19)	0.34464 (12)	0.22372 (18)	0.0225 (4)	
H15	0.2771	0.3444	0.1777	0.027*	
C16	0.09264 (19)	0.29929 (12)	0.16861 (18)	0.0240 (4)	
H16	0.0834	0.2680	0.0857	0.029*	
C17	−0.00860 (19)	0.30017 (12)	0.23605 (18)	0.0227 (4)	
H17	−0.0876	0.2691	0.1993	0.027*	
C18	0.00458 (17)	0.34619 (12)	0.35696 (17)	0.0190 (4)	
H18	−0.0655	0.3464	0.4023	0.023*	

### Atomic displacement parameters (Å^2^)

**Table:**

	*U*^11^	*U*^22^	*U*^33^	*U*^12^	*U*^13^	*U*^23^
C1	0.0136 (8)	0.0170 (8)	0.0167 (8)	0.0015 (7)	0.0033 (6)	0.0001 (6)
N1	0.0168 (7)	0.0203 (8)	0.0156 (7)	−0.0023 (6)	0.0049 (5)	0.0008 (6)
N2	0.0190 (7)	0.0188 (7)	0.0145 (7)	−0.0030 (6)	0.0049 (6)	0.0001 (6)
N3	0.0158 (7)	0.0209 (8)	0.0153 (7)	−0.0029 (6)	0.0053 (6)	0.0002 (6)
C2	0.0181 (9)	0.0161 (8)	0.0156 (8)	−0.0008 (7)	0.0062 (7)	−0.0028 (6)
O1	0.0167 (6)	0.0247 (7)	0.0217 (6)	0.0005 (5)	0.0058 (5)	0.0048 (5)
C3	0.0200 (9)	0.0151 (8)	0.0175 (8)	0.0000 (7)	0.0086 (7)	−0.0019 (6)
C4	0.0210 (9)	0.0198 (9)	0.0201 (9)	−0.0001 (7)	0.0059 (7)	0.0017 (7)
C5	0.0213 (9)	0.0243 (10)	0.0308 (10)	−0.0031 (8)	0.0098 (8)	0.0006 (8)
C6	0.0304 (10)	0.0207 (9)	0.0291 (10)	−0.0032 (8)	0.0180 (8)	0.0001 (8)
C7	0.0332 (11)	0.0197 (9)	0.0177 (8)	0.0006 (8)	0.0101 (8)	0.0015 (7)
C8	0.0221 (9)	0.0189 (9)	0.0181 (8)	0.0001 (7)	0.0060 (7)	−0.0009 (7)
C9	0.0272 (10)	0.0227 (9)	0.0168 (8)	−0.0064 (8)	0.0053 (7)	−0.0036 (7)
C10	0.0258 (10)	0.0346 (11)	0.0234 (9)	−0.0083 (9)	0.0011 (8)	−0.0010 (8)
C11	0.0228 (9)	0.0232 (9)	0.0146 (8)	−0.0021 (7)	0.0041 (7)	0.0041 (7)
C12	0.0269 (10)	0.0337 (11)	0.0205 (9)	−0.0046 (8)	0.0105 (8)	0.0014 (8)
C13	0.0176 (8)	0.0139 (8)	0.0145 (8)	0.0016 (7)	0.0022 (6)	0.0020 (6)
C14	0.0167 (9)	0.0217 (9)	0.0202 (9)	−0.0014 (7)	0.0044 (7)	−0.0008 (7)
C15	0.0231 (9)	0.0224 (9)	0.0242 (9)	0.0018 (7)	0.0102 (7)	−0.0009 (7)
C16	0.0339 (11)	0.0197 (9)	0.0183 (9)	−0.0010 (8)	0.0056 (8)	−0.0035 (7)
C17	0.0248 (10)	0.0193 (9)	0.0225 (9)	−0.0042 (7)	0.0023 (7)	−0.0011 (7)
C18	0.0172 (9)	0.0200 (9)	0.0201 (8)	−0.0007 (7)	0.0049 (7)	0.0022 (7)

### Geometric parameters (Å, °)

**Table:**

C1—N1	1.336 (2)	C9—H9A	0.9900
C1—N2	1.340 (2)	C9—H9B	0.9900
C1—N3	1.370 (2)	C10—H10A	0.9800
N1—C2	1.352 (2)	C10—H10B	0.9800
N2—C9	1.469 (2)	C10—H10C	0.9800
N2—C11	1.474 (2)	C11—C12	1.524 (3)
N3—C13	1.414 (2)	C11—H11A	0.9900
N3—H3	0.90 (2)	C11—H11B	0.9900
C2—O1	1.247 (2)	C12—H12A	0.9800
C2—C3	1.506 (2)	C12—H12B	0.9800
C3—C8	1.396 (2)	C12—H12C	0.9800
C3—C4	1.397 (3)	C13—C14	1.397 (3)
C4—C5	1.388 (3)	C13—C18	1.400 (2)
C4—H4	0.9500	C14—C15	1.389 (3)
C5—C6	1.388 (3)	C14—H14	0.9500
C5—H5	0.9500	C15—C16	1.386 (3)
C6—C7	1.388 (3)	C15—H15	0.9500
C6—H6	0.9500	C16—C17	1.385 (3)
C7—C8	1.392 (3)	C16—H16	0.9500
C7—H7	0.9500	C17—C18	1.388 (3)
C8—H8	0.9500	C17—H17	0.9500
C9—C10	1.519 (3)	C18—H18	0.9500
			
N1—C1—N2	118.14 (15)	C9—C10—H10A	109.5
N1—C1—N3	124.62 (15)	C9—C10—H10B	109.5
N2—C1—N3	117.14 (15)	H10A—C10—H10B	109.5
C1—N1—C2	121.38 (15)	C9—C10—H10C	109.5
C1—N2—C9	119.52 (14)	H10A—C10—H10C	109.5
C1—N2—C11	124.62 (15)	H10B—C10—H10C	109.5
C9—N2—C11	115.71 (14)	N2—C11—C12	113.04 (15)
C1—N3—C13	126.83 (15)	N2—C11—H11A	109.0
C1—N3—H3	117.8 (15)	C12—C11—H11A	109.0
C13—N3—H3	114.9 (14)	N2—C11—H11B	109.0
O1—C2—N1	127.02 (16)	C12—C11—H11B	109.0
O1—C2—C3	118.52 (16)	H11A—C11—H11B	107.8
N1—C2—C3	114.36 (15)	C11—C12—H12A	109.5
C8—C3—C4	119.10 (16)	C11—C12—H12B	109.5
C8—C3—C2	119.54 (16)	H12A—C12—H12B	109.5
C4—C3—C2	121.33 (16)	C11—C12—H12C	109.5
C5—C4—C3	120.41 (17)	H12A—C12—H12C	109.5
C5—C4—H4	119.8	H12B—C12—H12C	109.5
C3—C4—H4	119.8	C14—C13—C18	118.86 (16)
C6—C5—C4	120.22 (18)	C14—C13—N3	123.74 (16)
C6—C5—H5	119.9	C18—C13—N3	117.28 (16)
C4—C5—H5	119.9	C15—C14—C13	119.85 (17)
C5—C6—C7	119.79 (17)	C15—C14—H14	120.1
C5—C6—H6	120.1	C13—C14—H14	120.1
C7—C6—H6	120.1	C16—C15—C14	121.16 (18)
C6—C7—C8	120.23 (17)	C16—C15—H15	119.4
C6—C7—H7	119.9	C14—C15—H15	119.4
C8—C7—H7	119.9	C17—C16—C15	119.13 (17)
C7—C8—C3	120.24 (17)	C17—C16—H16	120.4
C7—C8—H8	119.9	C15—C16—H16	120.4
C3—C8—H8	119.9	C16—C17—C18	120.46 (17)
N2—C9—C10	113.05 (16)	C16—C17—H17	119.8
N2—C9—H9A	109.0	C18—C17—H17	119.8
C10—C9—H9A	109.0	C17—C18—C13	120.53 (17)
N2—C9—H9B	109.0	C17—C18—H18	119.7
C10—C9—H9B	109.0	C13—C18—H18	119.7
H9A—C9—H9B	107.8		
			
N2—C1—N1—C2	−141.68 (17)	C5—C6—C7—C8	0.5 (3)
N3—C1—N1—C2	42.2 (3)	C6—C7—C8—C3	0.1 (3)
N1—C1—N2—C9	10.7 (2)	C4—C3—C8—C7	−0.7 (3)
N3—C1—N2—C9	−172.82 (15)	C2—C3—C8—C7	177.63 (16)
N1—C1—N2—C11	−164.64 (16)	C1—N2—C9—C10	−85.7 (2)
N3—C1—N2—C11	11.8 (2)	C11—N2—C9—C10	90.06 (19)
N1—C1—N3—C13	22.4 (3)	C1—N2—C11—C12	−110.70 (19)
N2—C1—N3—C13	−153.84 (16)	C9—N2—C11—C12	73.8 (2)
C1—N1—C2—O1	17.6 (3)	C1—N3—C13—C14	19.1 (3)
C1—N1—C2—C3	−166.17 (15)	C1—N3—C13—C18	−164.97 (16)
O1—C2—C3—C8	−12.0 (2)	C18—C13—C14—C15	1.1 (3)
N1—C2—C3—C8	171.40 (16)	N3—C13—C14—C15	176.97 (16)
O1—C2—C3—C4	166.25 (16)	C13—C14—C15—C16	−0.9 (3)
N1—C2—C3—C4	−10.3 (2)	C14—C15—C16—C17	0.2 (3)
C8—C3—C4—C5	0.8 (3)	C15—C16—C17—C18	0.3 (3)
C2—C3—C4—C5	−177.53 (16)	C16—C17—C18—C13	−0.1 (3)
C3—C4—C5—C6	−0.2 (3)	C14—C13—C18—C17	−0.6 (3)
C4—C5—C6—C7	−0.4 (3)	N3—C13—C18—C17	−176.78 (16)

### Hydrogen-bond geometry (Å, °)

**Table:**

*D*—H···*A*	*D*—H	H···*A*	*D*···*A*	*D*—H···*A*
N3—H3···O1^i^	0.90 (2)	1.97 (2)	2.852 (2)	168 (2)

Symmetry codes: (i) −*x*, −*y*+1, −*z*+1.
